# Genetic variation and genetic structure within metapopulations of two closely related selfing and outcrossing *Zingiber* species (Zingiberaceae)

**DOI:** 10.1093/aobpla/plaa065

**Published:** 2020-12-02

**Authors:** Rong Huang, Zong-Dian Zhang, Yu Wang, Ying-Qiang Wang

**Affiliations:** 1 Guangdong Provincial Key Laboratory of Biotechnology for Plant Development, School of Life Sciences, South China Normal University, Guangzhou, China; 2 Guangzhou Key Laboratory of Subtropical Biodiversity and Biomonitoring, School of Life Sciences, South China Normal University, Guangzhou, China

**Keywords:** Fragmentation, genetic differentiation, mating system, *Zingiber corallinum*, *Zingiber nudicarpum*

## Abstract

Habitat fragmentation strongly affects the genetic diversity of plant populations, and this has always attracted much research interest. Although numerous studies have investigated the effects of habitat fragmentation on the genetic diversity of plant populations, fewer studies have compared species with contrasting breeding systems while accounting for phylogenetic distance. Here, we compare the levels of genetic diversity and differentiation within and among subpopulations in metapopulations (at fine-scale level) of two closely related *Zingiber* species, selfing *Zingiber corallinum* and outcrossing *Zingiber nudicarpum*. Comparisons of the genetic structure of species from unrelated taxa may be confounded by the effects of correlated ecological traits or/and phylogeny. Thus, we possibly reveal the differences in genetic diversity and spatial distribution of genetic variation within metapopulations that relate to mating systems. Compared to outcrossing *Z*. *nudicarpum*, the subpopulation genetic diversity in selfing *Z*. *corallinum* was significantly lower, but the metapopulation genetic diversity was not different. Most genetic variation resided among subpopulations in selfing *Z*. *corallinum* metapopulations, while a significant portion of variation resided either within or among subpopulations in outcrossing *Z*. *nudicarpum*, depending on whether the degree of subpopulation isolation surpasses the dispersal ability of pollen and seed. A stronger spatial genetic structure appeared within subpopulations of selfing *Z*. *corallinum* potentially due to restricted pollen flow and seed dispersal. In contrast, a weaker genetic structure was apparent in subpopulations of outcrossing *Z*. *nudicarpum* most likely caused by extensive pollen movement. Our study shows that high genetic variation can be maintained within metapopulations of selfing *Zingiber* species, due to increased genetic differentiation intensified primarily by the stochastic force of genetic drift among subpopulations. Therefore, maintenance of natural variability among subpopulations in fragmented areas is key to conserve the full range of genetic diversity of selfing *Zingiber* species. For outcrossing *Zingiber* species, maintenance of large populations is an important factor to enhance genetic diversity.

Compared to outcrossing *Z. nudicarpum*, the subpopulation genetic diversity in selfing *Z. corallinum* was significantly lower, but the metapopulation genetic diversity did not differ. Most genetic variation resided among subpopulations in selfing *Z. corallinum* metapopulations, while a significant portion of variation resided either within or among subpopulations in outcrossing *Z. nudicarpum*, depending on whether the degree of subpopulation isolation surpasses the dispersal ability of pollen and seed. Our study shows that selfing *Z. corallinum* could maintain high genetic diversity through differentiation intensified primarily by the stochastic force of genetic drift among subpopulations at fine-scale level, but not local adaptation.

## Introduction

Genetic diversity is central to the maintenance of biodiversity in any ecosystem and allows for adaptive evolution in changing environments (Govindaraj *et al.* 2015). Plant mating systems have a marked impact on the magnitude of genetic variability (Williams *et al.* 2001). For example, compared with outcrossing, selfing species may exhibit lower effective population size and recombination rates, leading to reduced polymorphism (diversity), increased linkage disequilibrium and increased homozygosity (Glémin *et al.* 2006; López-Villalobos and Eckert 2018). Indeed, numerous studies comparing selfing and outcrossing species regarding the genetic diversity of their populations support decreased genetic diversity within populations of selfing species (Ingvarsson 2002; Eschmann-Grupe *et al.* 2004; Nybom 2004; Segarra-Moragues and Mateu-Andrés 2007; Reisch and Bernhardt-Römermann 2014; Pettengill *et al.* 2016). Nevertheless, other studies have shown that selfing species can have low levels of genetic variation within populations, while still maintaining high levels of genetic diversity at the metapopulation level (Govindaraju 1988; Whitlock and Barton 1997; Pannell and Charlesworth 2000). Thus, metapopulation dynamics may affect genetic structure and the level of population diversity and differentiation (Céspedes *et al.* 2003; Settepani *et al.* 2014). To date, however, the effects of metapopulation dynamics on plant population genetic diversity and genetic structure are ambiguous, because of the apparent lack of a uniform genetic response to habitat fragmentation (Berge *et al.* 1998; Galeuchet *et al.* 2005).

The dramatically increasing fragmentation of natural habitats as a result of anthropic activities is markedly affecting the genetic diversity of plant populations and has been the subject of research interest (Young *et al.* 1996; Samarasin *et al.* 2017). Theoretically, habitat fragmentation can cause a loss of genetic variation in a population due to increased random genetic drift, inbreeding and reduced gene flow (Young *et al.* 1996; Lowe *et al.* 2005). However, a complex set of factors that include mating system, pollination mechanism, seed dispersal, geographic range and the genetic basis of inbreeding depression may influence the degree of susceptibility to fragmentation (Aguilar *et al.* 2006). Of these, the mating system is one of the main attributes influencing vulnerability to fragmentation effects (Aizen *et al.* 2002). Honnay and Jacquemyn (2007) concluded that outcrossing species were more vulnerable than selfing species to the loss of genetic variation through habitat fragmentation. The role of gene flow in maintaining genetic diversity in outbreeding species is very important; it can maintain a high degree of genetic diversity in a population by frequent exchange of genes with other populations and even a very few migrants per generation are sufficient to counter genetic differentiation (Wright 1931; Jacquemyn *et al.* 2007; Ohbayashi *et al.* 2017). Aguilar *et al.* (2008) also suggested that mixed-mating and selfing species suffer fewer losses of alleles and polymorphic loci than self-incompatible and outcrossing species in fragmented populations by reduced gene flow. Gene flow together with the type of mating system ultimately determine the spatial pattern of gene movement within and among plant populations (Nathan and Muller-Landau 2000).

In most plants, there are at least two possible carriers of genes, i.e. pollen and seed, which may have different potential for dispersal, and thus different consequences for population structure (Grivet *et al.* 2009). Gene flow in seed plants is a two-step process via pollen and then by seed, and the observed genetic structure of a population may be more strongly influenced by limited seed dispersal than by pollen movement (Chung *et al.* 2000), since seeds carry two copies of gene, while pollen carries one copy. However, pollen dispersal has usually more potential for long-distance gene transport by wind or animal pollinator, and thus contributes an important component of total gene flow (McCauley 1997; Latta *et al.* 1998; Oddou-Muratorio *et al.* 2005, 2006). Pollen movement and seed dispersal greatly affect genetic processes that can have a major impact on evolution, such as ‘isolation by distance’ within continuous populations (Robledo-Arnuncio and Gil 2005) and gene exchange among population fragments (Bacles and Ennos 2008). Outcrossing species always tend to generate less spatial genetic structure than selfing species (Vekemans and Hardy 2004) due to a higher degree of gene flow via pollen (Duminil *et al.* 2009). On the other hand, fine-scale genetic structure may appear in species with extensive pollen movement but restricted seed dispersal, such as *Araucaria angustifolia* (Bittencourt and Sebbenn 2007), *Quercus lobata* (Grivet *et al.* 2009) and *Theobroma cacao* (Silva *et al.* 2011). Up to date, most studies on genetic diversity within metapopulations have focused on outcrossing plants (Bacles *et al.* 2004; Bittencourt and Sebbenn 2007; Bacles and Ennos 2008; Born *et al.* 2008; Wang *et al.* 2011; Cascante-Marín *et al.* 2014), with less attention being paid to selfing plants (Bonnin *et al.* 2001; Volis *et al.* 2010, 2016). Moreover, as far as we are aware, there have been few studies on the effect of fragmentation comparing closely related selfing and outcrossing species of the same genus. Comparisons of the genetic structure of species from unrelated taxa may be confounded by the effects of correlated ecological traits or/and phylogeny (Williams *et al.* 2001). Thus, comparisons of closely related species with different mating systems could contribute to an improved understanding of the effect of mating system on population genetic diversity and structure (Charlesworth 2003).

A previous study on comparing the genetic structure of two closely related selfing and outcrossing *Zingiber* species within the same geographic area (at landscape level) showed that selfing *Z*. *corallinum* can maintain a high level of genetic diversity across the species’ range, similar to that of outcrossing *Z*. *nudicarpum*, albeit low genetic diversity within populations (Huang *et al.* 2019). Factors influencing genetic structure are different at various level (Jones and Hubbell 2006; Ouinsavi *et al.* 2009). Historical factors and isolation by distance, at landscape-level scales, have the greatest influence on population genetic structure (Ouinsavi *et al.* 2009), and fine-scale genetic structure has been attributed to seed and pollen dispersal (Jones and Hubbell 2006). Thus, knowledge of genetic structure at different scales (e.g. landscape levels and fine-scale levels) is crucial for evaluating importance of evolutionary processes and defining important strategies for conservation genetics (Vieira *et al.* 2010). In this study, we compare the levels of genetic variation and differentiation within and among subpopulations in metapopulations (at fine-scale level) of two closely related *Zingiber* species with identical seed dispersal mode, selfing *Zingiber corallinum* and outcrossing *Zingiber nudicarpum*, using ISSR (inter-simple sequence repeat) data. The ISSR is an easy handling, good reproducibility, low cost, quick technique for plant population genetic studies (Tabina *et al.* 2016). Although ISSR markers are dominant (Bentley *et al.* 2015), formulae that estimate heterozygosity are applicable to this type of data (Nei 1973; Mariette *et al.* 2002). We aim to address the following, at fine-scale level: (i) whether selfing *Z*. *corallinum* show less genetic diversity than outcrossing *Z*. *nudicarpum* within metapopulations as theory predicts; or (ii) whether selfing *Z*. *corallinum* also can maintain high genetic diversity within metapopulations, like that at landscape level; (iii) whether there are differences in the spatial distribution of genetic variation among *Zingiber* metapopulations that relate to mating system and gene flow?

## Materials and Methods

### Species, study sites and sample collection

**Figure 1.**
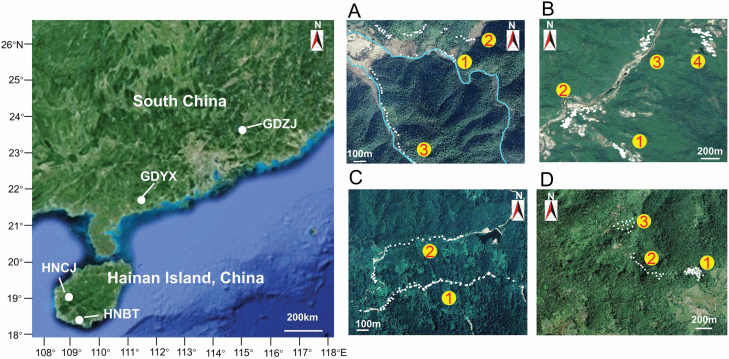
Location of the metapopulations and distribution of subpopulations in metapopulations of *Zingiber corallinum* (A—GDZJ; B—GDYX) and *Z. nudicarpum* (C—HNCJ; D—HNBT). Each number (1–4) represents one subpopulation while each dot represents a sampled individual, and blue lines indicate streams. The map was drawn by the authors with reference to Google Maps (https://maps.google.com/).

Both species of *Zingiber*, *Z*. *corallinum* and *Z*. *nudicarpum*  **[see**  **Supporting Information—Fig. S1****]** are from the same section Zingiber of the genus *Zingiber* (Wu 1981; Fang 1982; Wu and Larsen 2000). Both species are diploid perennial herbs with hermaphrodite flowers and distribute in Guangdong, Guangxi and Hainan in south China. *Zingiber corallinum* flowers in May to August and is mainly self-pollinated by the bending of stigma towards the anther, with 74.7 % auto-fertility index (Wu 2008). *Zingiber nudicarpum* flowers in April to June and is usually cross-pollinated by a parasitic bee, without autonomous selfing mode (Tan 2010). Primary seed dispersal of both species takes place by gravity (unpubl. data). For each species, two metapopulations with different degrees of fragmentation were studied. Here, we define a metapopulation as a subset of plant subpopulations separated by ca. 200–1500 m with potential for exchange of a propagules (i.e. pollen and/or seeds) and a subpopulation as a community of potentially interbreeding individuals that live in the same habitat patch (Fig. 1). The two metapopulations of *Z*. *corallinum* (separated by over 400 km) are located in Zijin County (GDZJ—23°43′57″–23°43′59″N, 115°00′47″–115°00′51″E, alt. 396–427 m) and Yangxi County (GDYX—21°47′28″–21°47′32″N, 111°25′43″–111°26′26″E, alt. 215–272 m), Guangdong Province, China (Fig. 1). The two metapopulations of *Z*. *nudicarpum* (separated by nearly 100 km) are located in Baoting County (HNBT—18°25′00″–18°25′01″N, 109°33′32″–109°33′34″E, alt. 404–435 m) and Changjiang County (HNCJ—19°07′30″–19°07′32″N, 109°04′52″–109°05′08″E, alt. 562–711 m), Hainan Province, China (Fig. 1). The GDZJ metapopulation of *Z*. *corallinum* consists of three subpopulations, which are isolated by average ca. 300 m of agricultural land, village, stream or mountain forest. The plants grow in valleys on forest margins alongside a stream or on a hillside alongside mountain trails. At the GDYX site, the species has spatially structured populations with four subpopulations isolated by average ca. 859 m of farmland, village, mountain forest or stream. The individuals grow on open bamboo forestland near a village, farmland and alongside a stream. The HNCJ metapopulation of *Z*. *nudicarpum* consists of two subpopulations naturally separated by average ca. 725 m of mountain forest; the individuals grow on the margins of a mountain forest along roads. At the HNBT site, the species also has spatially structured populations with three subpopulations scattered widely in a more or less continuous area of suitable habitat (separated by average ca. 320 m apart). The individual plants are scattered within abandoned farmlands and the margins of remnant forest.

To investigate within-population spatial genetic structure, we collected samples from 15–74 adult individuals (at least 2 m apart) throughout the area of distribution of each subpopulation (ca. 20–83 mature individuals per subpopulation size) in metapopulations of *Z*. *corallinum* and *Z*. *nudicarpum* (Table 1). The straight-line distance between individuals was also estimated directly on the basis of the site coordinates to test the spatial autocorrelation coefficient (*r*) within subpopulations. In order to test the genetic correlation between seedlings and mature individuals and further analyse the spatial genetic structure within subpopulations, we also sampled 45 seedlings throughout the area of distribution of subpopulation 2 in the GDZJ metapopulation of *Z*. *corallinum*. Because seedlings always aggregated around adjacent mature individuals, we classified them into five sets with their nearest adult individuals (Set-M1 to Set-M5). Leaf tissue samples were stored in silica gel for DNA analysis.

**Table 1.** Genetic diversity parameters based on ISSR for metapopulations of *Zingiber corallinum* and *Z. nudicarpum*. Subpop. size: number of adult plants per subpopulation; PL: number of polymorphic loci; PPL: percentage of polymorphic loci; *N*_a_: number of observed alleles; *N*_e_: number of effective alleles; *h*: Nei’s gene diversity; *I*: Shannon’s information index; NS: number of specific bands.

Species	Metapop.	Subpop.	Subpop. size	Sample size	PL	PPL (%)	*N*_a_	*N*_e_	*h*	*I*	NS
*Z*. *corallinum*	GDZJ	1	34	26	39	19.8	1.1980	1.1289	0.0739	0.1091	12
		2	64	39	112	56.9	1.5685	1.3395	0.1986	0.2971	8
		3	68	31	33	16.8	1.1675	1.1185	0.0666	0.0969	16
		Average	55.3	32.0	61.3	31.1	1.3113	1.1956	0.1130	0.1677	12
		Total	166	96	183	92.9	1.7766	1.4425	0.2597	0.3904	36
	GDYX	1	42	32	21	12.0	1.1200	1.0259	0.0180	0.0315	4
		2	48	36	10	5.7	1.0571	1.0176	0.0107	0.0174	18
		3	32	24	36	20.6	1.2057	1.0971	0.0597	0.0922	14
		4	56	43	18	10.3	1.1029	1.0630	0.0356	0.0525	7
		Average	44.5	33.8	21.3	12.1	1.1214	1.0509	0.0310	0.0484	10.8
		Total	178	135	132	75.4	1.7543	1.4064	0.2383	0.3601	43
	Mean	Subpop.	49.1	33.0	38.4	20.3	1.2028	1.1129	0.0662	0.0995	11.3
		Metapop.	172	115.5	157.5	84.2	1.7655	1.4245	0.2490	0.3753	39.5
*Z*. *nudicarpum*	HNCJ	1	83	74	182	70.5	1.7054	1.2452	0.1572	0.2524	8
		2	39	31	133	51.6	1.5155	1.2447	0.1472	0.2257	17
		Average	61.0	52.5	157.5	61.1	1.6105	1.2450	0.1522	0.2391	12.5
		Total	122	105	242	93.8	1.9380	1.3929	0.2360	0.3708	25
	HNBT	1	64	37	128	64.3	1.6432	1.3151	0.1897	0.2907	0
		2	20	15	90	45.2	1.4523	1.2463	0.1434	0.2160	0
		3	28	16	62	31.2	1.3116	1.1593	0.0944	0.1438	0
		Average	37.3	22.7	93.3	46.9	1.4690	1.2402	0.1425	0.2168	0
		Total	102	68	147	73.9	1.7387	1.3607	0.2132	0.3252	0
	Mean	Subpop.	46.8	34.6	119	52.6	1.5256	1.2421	0.1464	0.2257	5
		Metapop.	112	86.5	194.5	83.8	1.8384	1.3768	0.2246	0.3480	12.5

### DNA extraction and PCR

DNA from the sampled leaves was extracted using the modified CTAB method (Doyle and Doyle 1987). The DNA concentration and quality were assessed using a spectrophotometer and 0.8 % agarose gel electrophoresis. ISSR–PCR amplifications were performed in a Bio-Rad T100 thermal cycler with the following profile: pre-denaturation at 95 °C for 5 min, followed by 39 cycles of denaturation at 94 °C for 45 s, annealing for 45 s and extension at 72 °C for 90 s, with a final extension at 72 °C for 10 min. Ten primers, selected from 64 ISSR primers used previously in references for Zingiberaceae **[see**  **Supporting Information—Table S1****]**, were used in *Z*. *corallinum*; the same 10 primers were also used in *Z*. *nudicarpum*, together with additional three primers for this species **[see**  **Supporting Information—Table S2****]**. PCR for both species was carried out in a total volume of 20 μL, including 40 ng template DNA, 2.5/2.0 μL 10× buffer, 1.50/1.00 mmol Mg^2+^, 0.15/0.20 mmol dNTPs, 0.4/0.6 μmol primer, 2.0 units of *Taq* DNA polymerase and double-distilled water. Negative controls, that replaced template DNA with distilled water, were included in each PCR set to test for possible contamination. PCR products were subjected to electrophoresis in 1.8 % agarose gels in 0.5× TBE buffer at 130 V for 1–1.5 h along with a 100-bp ladder and photographed with the help of a gel documentation system (Bio-Rad GelDoc XR^+^). To ensure reliability of the bands, duplicate PCR amplifications were performed and only clear and reproducible bands were scored. The images of the gels were analysed using Image Lab software (Bio-Rad) to score for the presence (1) or absence (0) of bands and to assign a fragment size to each band. The presence or absence of bands was further visually confirmed.

### Data analysis

#### Analysis of genetic diversity, differentiation and gene flow.

 The presence/absence binary matrix of the ISSR phenotypes was analysed using POPGENE v. 1.31 (Yeh *et al.* 1997) to estimate the genetic diversity parameters at subpopulation and metapopulation level as follows: percentage of polymorphic loci (PPL), Nei’s gene diversity (*h*), Shannon’s information index (*I*), observed number of alleles (*N*_a_), effective number of alleles (*N*_e_). Genetic differentiation among subpopulations was evaluated by *G*_ST_ in POPGENE. The matrix was also subjected to an analysis of molecular variance (AMOVA) using GenAlEx 6.502 (Peakall and Smouse 2012) based on 999 permutations to calculate the values of population genetic differentiation (Φ) and the proportion of total variation among and within subpopulations. Gene flow (*N*_m_) among subpopulations was estimated using the formula, *N*_m_ = 0.5(1 − *G*_st_)/*G*_st_ (McDermott and McDonald 1993). Genetic diversity parameters (*h* and *I*) of two species were statistically analysed by the independent *t*-test using SPSS software (SPSS Inc., Chicago, IL, USA).

#### Analysis of genetic structure.

Genetic cluster analysis implemented in InStruct v. 1.0 (Gao *et al.* 2007) for selfing *Z. corallinum* and STRUCTURE v. 2.1 (Pritchard *et al.* 2000) for outcrossing *Z. nudicarpum*, respectively, were used to infer the number of genetic units within metapopulations. We performed five runs with a burn-in length of 100 000 and a run length of 1 000 000 Markov chain Monte Carlo (MCMC) replications. The optimal value of *K* was calculated according to the method of Evanno *et al.* (2005). To visualize the genetic relationships between individuals, the genetic distance matrix was subjected to a principal coordinate analysis (PCoA) using GenAlEx.

#### Spatial genetic structure within subpopulations.

The genetic relatedness of individuals relative to their spatial position within four subpopulations in metapopulation GDYX and two subpopulations in metapopulation HNCJ was analysed by spatial autocorrelation. The autocorrelation coefficient (*r*) generated is a proper correlation coefficient, bounded by [–1, +1] and is closely related to Moran’s *I*. The spatial genetic structure of the studied subpopulations was tested using single population spatial structure analyses in GenAlEx. The even sample classes were chosen because this was particularly useful for reducing noisy confidence limits when sample sizes were very uneven (Peakall and Smouse 2012). Two-tailed probability values were calculated and bootstrap resampling was performed 999 times. To reveal the genetic relationship between seedlings and mature individuals, NTSYSpc-2.10 (Rohlf 2000) was used to conduct an unweighted pair-group method using an arithmetic average analysis (UPGMA) based on the Dice coefficient. We used POPGENE to calculate pairwise Nei’s genetic distances (Nei and Li 1979) between seedlings and mature individuals and a dendrogram was generated in MEGA v. 7 (Kumar *et al.* 2016) from the genetic distances matrix using the neighbour-joining (NJ) algorithm. Mantel tests implemented in GenAlEx were also performed to analyse the effects of geographical distance on genetic structure between subpopulations.

## Results

### ISSR polymorphism and genetic diversity

The primers produced 221 and 300 reliable ISSR bands from seven and five subpopulations in the metapopulations of *Z*. *corallinum* and *Z*. *nudicarpum*, respectively, of which 215 (97.3 %) and 294 (98.0 %) were polymorphic. The size of bands ranged from 200 to 2150 bp and 210 to 1900 bp, respectively. Of these, there were 197 and 175 reliable ISSR bands in metapopulations GDZJ and GDYX of *Z*. *corallinum*, of which 183 (92.9 %) and 132 (75.4 %) were polymorphic, respectively, and the specific bands were 36 (18.3 %) and 43 (24.6 %), average 39.5 (21.4 %) (Table 1). At the metapopulation level, the averages of *h* and *I* were 0.2490 and 0.3753 for *Z*. *corallinum*. At the subpopulation level, *h* and *I* ranged from 0.0107 to 0.1986 (average 0.0662) and from 0.0174 to 0.2971 (average 0.0995), respectively (Table 1). For the HNCJ and HNBT metapopulations of *Z*. *nudicarpum*, 242 (93.8 %) and 147 (73.9 %) of the 258 and 199 reliable bands were polymorphic and 0–25 (0–9.7 %) specific bands were found, with an average of 12.5 (4.9 %) (Table 1). At the metapopulation level, the averages of *h* and *I* were 0.2246 and 0.3480 for *Z*. *nudicarpum*. At the subpopulation level, *h* and *I* ranged from 0.0944 to 0.1897 (average 0.1464), and from 0.1438 to 0.2907 (average 0.2257), respectively (Table 1).

**Figure 2.**
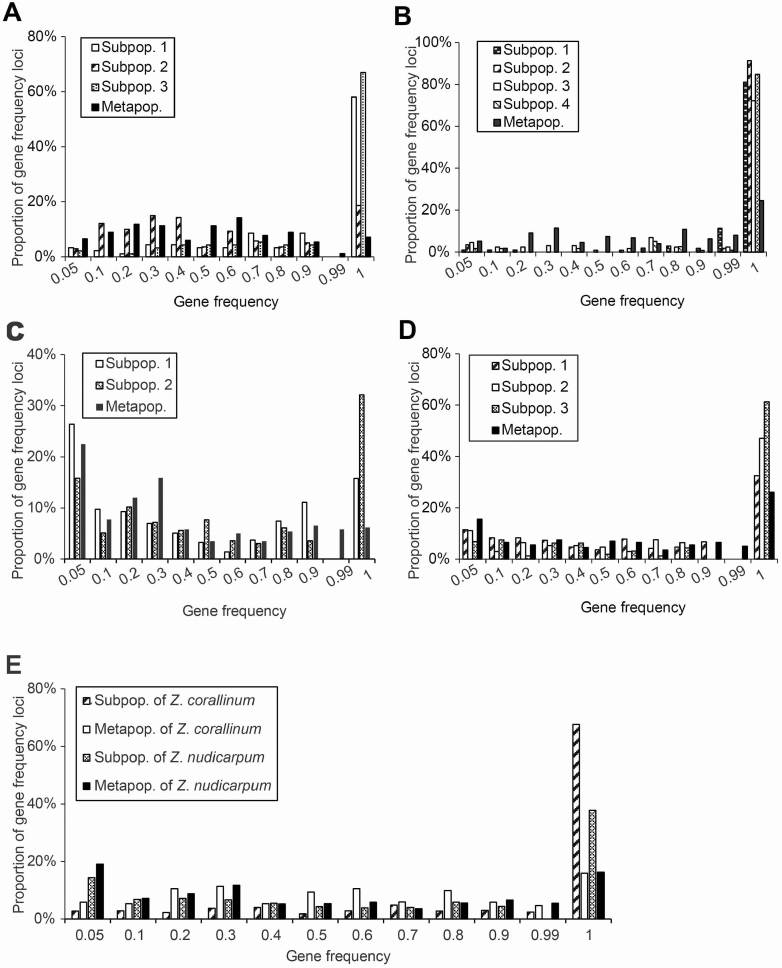
Distribution of gene frequency in metapopulations of *Zingiber corallinum* (A—GDZJ; B—GDYX) and *Z. nudicarpum* (C—HNCJ; D—HNBT) and their mean values (E).

Among all subpopulations in the two *Z. corallinum* metapopulations except GDZJ-2, common loci (i.e. found in all individuals/subpopulations: gene frequency = 100 %) accounted for the highest proportion of amplified fragments (58.1–91.4 %), while the medium–high gene frequency loci (50 % < gene frequency < 100 %) accounted for a higher proportion of amplified fragments (4.3–23.7 %) than the low–medium gene frequency loci (5 % < gene frequency ≤ 50 %) (0.7–15.1 %) (Fig. 2A and B). Rare loci (gene frequency ≤ 5 %) accounted for a very low proportion in all seven subpopulations (0.9–4.2 %) (Fig. 2A and B). At the metapopulation level, both common loci and rare loci of GDZJ and GDYX were less prevalent, i.e. 7.1 and 24.6 %, and 6.5 and 5.1 %, respectively, but loci with low–medium and medium–high gene frequency accounted for a higher proportion of amplified fragments, i.e. 49.1 and 34.3 %, and 37.3 and 36.0 %, respectively (Fig. 2A and B). Among all subpopulations in the two *Z*. *nudicarpum* metapopulations, low–medium gene frequency loci were more prevalent (23.1–35.7 %) than medium–high gene frequency loci (8.7–23.6 %). With the exception of HNCJ-1, common loci and rare loci in the remaining four *Z*. *nudicarpum* subpopulations accounted for the highest proportion (32.4–61.3 %) and the lowest proportion (6.9–15.8 %) of amplified fragments, respectively (Fig. 2C and D). At the metapopulation level, in both metapopulations (HNCJ and HNBT) low–medium gene frequency loci accounted for the highest proportion (45.0 and 31.2 %) of amplified fragments, followed by the medium–high gene frequency loci (26.4 and 27.1 %). However, there were proportionally more common loci (26.1 %) and fewer rare loci (15.6 %) in HNBT than in HNCJ, where common loci outnumbered rare loci (6.2 % vs. 22.5 %) (Fig. 2C and D).

### Genetic differentiation and gene flow among subpopulations within metapopulations

Nei’s *G*_st_ values for the subpopulations in *Z*. *corallinum* and *Z*. *nudicarpum* metapopulations were estimated as 0.7110 and 0.3408 (Table 2), respectively, which indicates that only 28.9 % and 65.9 %, respectively, of the genetic variability was distributed within subpopulations. The estimates of gene flow (*N*_m_) per generation among subpopulations in *Z*. *corallinum* and *Z*. *nudicarpum* metapopulations were 0.2575 and 1.0311, respectively (Table 2). The AMOVA analysis was consistent with Nei’s genetic differentiation statistics, showing that 78.4 % (Φ _ST_ = 0.784) and 46.8 % (Φ _ST_ = 0.468) of the total variation was partitioned among subpopulations in the metapopulations of *Z*. *corallinum* and *Z*. *nudicarpum*, respectively (Table 3). That is, of the total molecular variance, only 21.6 % was attributable to individual differences within subpopulations in selfing *Z*. *corallinum* metapopulations, but 53.2 % was attributable to individual differences in outcrossing *Z*. *nudicarpum*.

**Table 2.** Genetic differentiation statistics among subpopulations within metapopulations of *Zingiber corallinum* and *Z*. *nudicarpum*. *H*_T_: total population diversity; *H*_S_: average within-population diversity; *G*_st_: the population differentiation; *N*_m_: gene flow.

Species	Metapopulation	*H*_T_	*H*_S_	*G*_st_	*N*_m_
*Z*. *corallinum*	GDZJ	0.2499	0.1131	0.5475	0.4432
	GDYX	0.2469	0.0310	0.8745	0.0718
	Total	0.2484	0.0721	0.7110	0.2575
*Z*. *nudicarpum*	HNCJ	0.2582	0.1522	0.4105	0.7180
	HNBT	0.1955	0.1425	0.2711	1.3442
	Total	0.2269	0.1474	0.3408	1.0311

**Table 3.** Summary of molecular variance (AMOVA) for metapopulations of *Zingiber corallinum* and *Z*. *nudicarpum*. df: degree of freedom; Ф _ST_: among subpopulations deviations from Hardy–Weinberg expectations; *P*: the probability of null hypothesis.

Metapopulation	Source	df	Sums of squares	Mean squares	Variance component	Percentage of variation	Ф _ST_	*P*
*Z*. *corallinum*								
GDZJ	Among subpopulations	2	1475.266	737.633	23.008	66.3 %	0.663	0.001
	Within subpopulations	93	1086.526	11.683	11.683	33.7 %		
	Total	95	2561.792		34.691	100 %		
GDYX	Among subpopulations	3	2491.693	830.564	24.875	90.5 %	0.905	0.001
	Within subpopulations	131	343.359	2.621	2.621	9.5 %		
	Total	134	2835.052		27.496	100 %		
Mean	Among subpopulations				23.942	78.4 %	0.784	
	Within subpopulations				12.152	21.6 %		
	Total				31.094	100 %		
*Z. nudicarpum*								
HNCJ	Between subpopulations	1	1423.739	1423.739	32.142	62.5 %	0.625	0.001
	Within subpopulations	103	1985.061	19.272	19.272	37.5 %		
	Total	104	3408.800		51.415	100 %		
HNBT	Between subpopulations	2	305.869	152.934	6.759	31.0 %	0.310	0.001
	Within subpopulations	65	979.616	15.071	15.071	69.0 %		
	Total	67	1285.485		21.830	100 %		
Mean	Between subpopulations				19.451	46.8 %	0.468	
	Within subpopulations				17.172	53.2 %		
	Total				36.623	100 %		

### Genetic structure and cluster analysis within metapopulations

**Figure 3.**
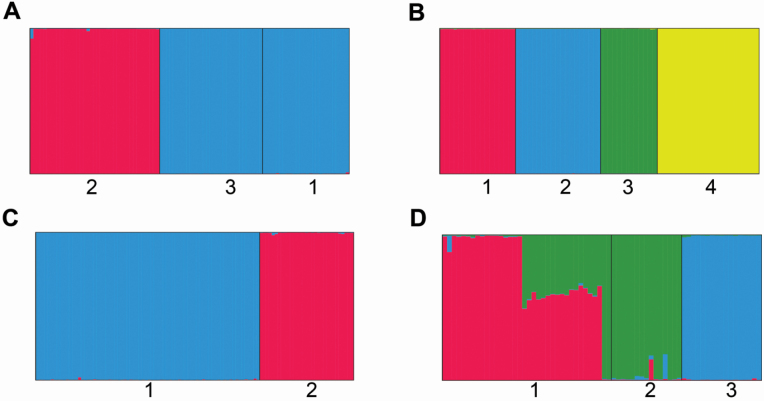
Metapopulations’ genetic group structure analysis by InStruct for *Zingiber corallinum* (A—GDZJ, *K* = 2; B—GDYX, *K* = 4) and STRUCTURE for *Z. nudicarpum* (C—HNCJ, *K* = 2; D—HNBT, *K* = 3). Each individual vertical bar represents an individual and the black vertical bars separate the subpopulations, while different colours represent different gene pools.

Genetic analyses performed with InStruct and STRUCTURE revealed that with the log likelihood reaching a maximum value at *K* = 2 and *K* = 4 in the two *Z*. *corallinum* metapopulations (GDZJ and GDYX, respectively), all subpopulations could be assigned to two and four genetic clusters (Fig. 3A and B). Within the GDZJ metapopulation, all individuals from subpopulations GDZJ-1 and GDZJ-3 were assigned to the same cluster and all individuals from subpopulation GDZJ-2 were assigned to a second cluster (Fig. 3A), while each of the four subpopulations of the GDYX metapopulation corresponded to a separate genetic cluster (Fig. 3B). Given the maximum log-likelihood values (*K* = 2 and *K* = 3; **see**  **Supporting Information—Fig. S2**), all subpopulations of the two *Z*. *nudicarpum* metapopulations (HNCJ and HNBT) were assigned into two and three genetic clusters (Fig. 3C and D), respectively, and all individuals within each subpopulation with the exception of HNBT-1 were assigned to the same genetic cluster. In subpopulation HNBT-1, there was a high degree of admixture of two gene pools in almost half the individuals, indicating possible crossbreeding events (Fig. 3D).

**Figure 4.**
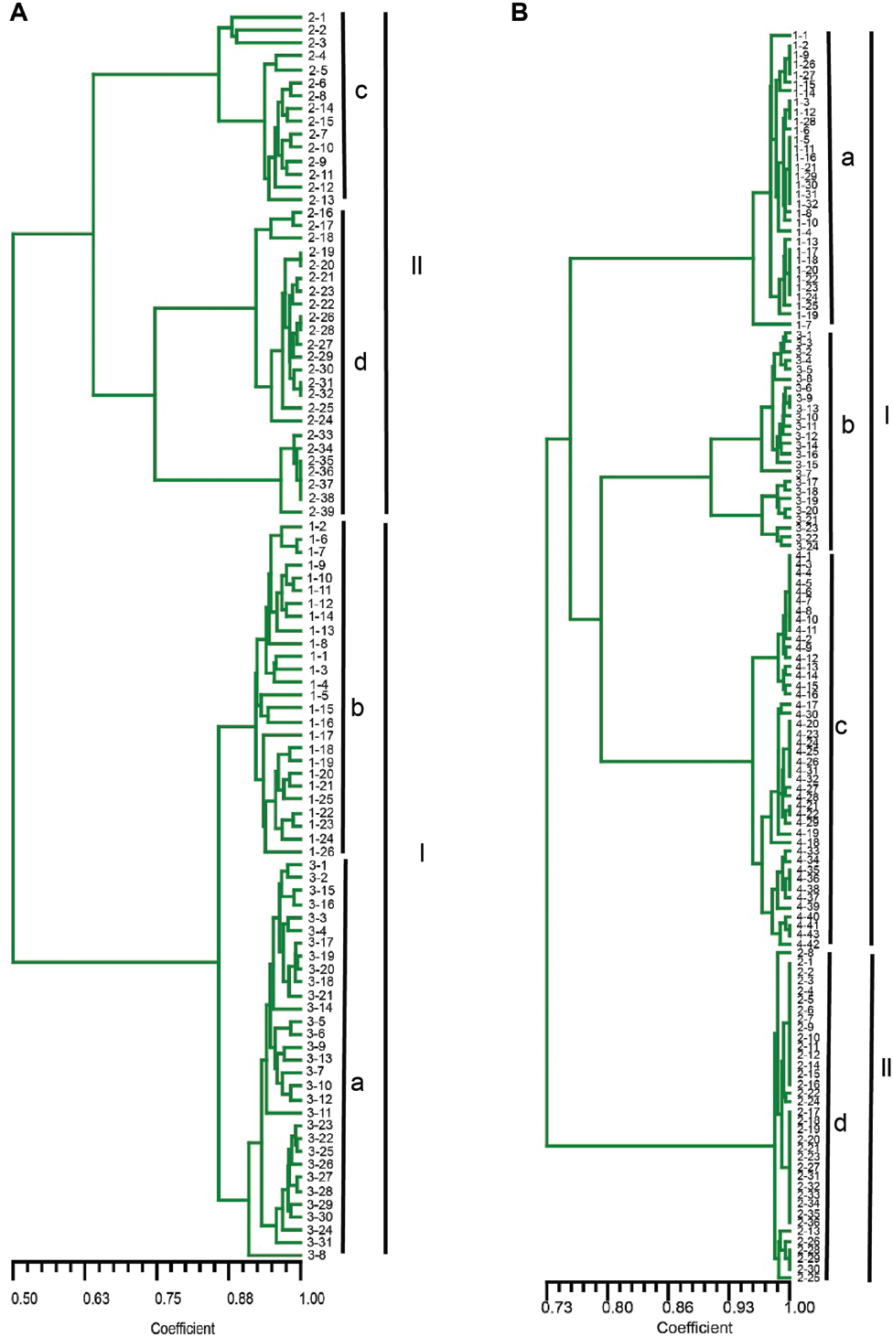
UPGMA dendrogram based on Dice coefficient for individuals in metapopulations of *Zingiber corallinum* (A—GDZJ; B—GDYX).

**Figure 5.**
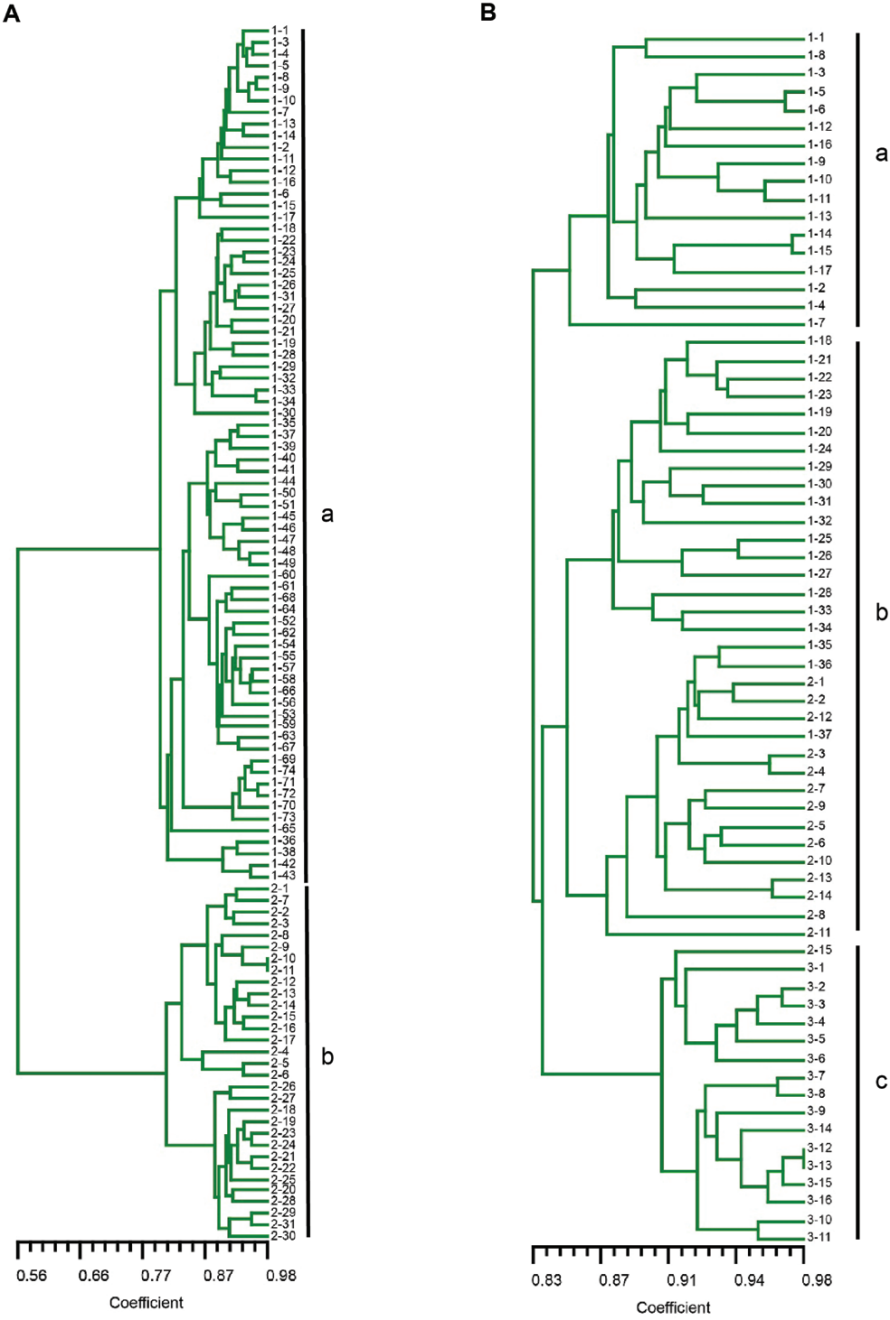
UPGMA dendrogram based on Dice coefficient for individuals in metapopulations of *Zingiber nudicarpum* (A—HNCJ; B—HNBT).

The UPGMA dendrogram (Figs 4 and 5) based on the Dice coefficient was consistent with an unrooted NJ tree **[see**  **Supporting Information—Figs S3**  **and**  **S4****]** based on Nei’s genetic distance in the four metapopulations of *Z*. *corallinum* and *Z*. *nudicarpum*. In the two metapopulations of selfing *Z*. *corallinum*, all individuals from the same subpopulations were clustered together. The 96 individuals from the GDZJ metapopulation were first grouped into two clusters (I, II) and then cluster I further formed two well-resolved clades (a, b), which comprised all individuals from subpopulations GDZJ-3 and GDZJ-1, respectively (Fig. 4A; **see**  **Supporting Information—Fig. S3A**). Cluster II comprised all individuals from subpopulation GDZJ-2, which also formed two further clades (c, d). The 135 individuals from metapopulation GDYX were also first grouped into two clusters (I, II) and cluster I further formed two groups with three well-resolved clades (a, b, c), which comprised all individuals from subpopulations GDYX-1, GDY-3 and GDYX-4, respectively (Fig. 4B; **see**  **Supporting Information—Fig. S3B**). Cluster II consisted of clade d only, which comprised all individuals from subpopulation GDYX-2. In the HNCJ metapopulation of outcrossing *Z*. *nudicarpum*, the 105 individuals were grouped into two clades (a, b) and all individuals from the same subpopulations clustered together (Fig. 5A; **see**  **Supporting Information—Fig. S4A**). The 68 individuals from metapopulation HNBT were grouped into three well-resolved clades (a, b, c), which comprised most individuals from subpopulations HNBT-1, HNBT-2 and HNBT-3, respectively (Fig. 5B; **see**  **Supporting Information—Fig. S4B**). That is, not all individuals from the same subpopulations of metapopulation HNBT were clustered together. Principal coordinate analysis confirmed the partitioning results of the UPGMA and NJ clustering **[see**  **Supporting Information—Fig. S5****]**.

### Spatial genetic structure within subpopulations and metapopulations

**Figure 6.**
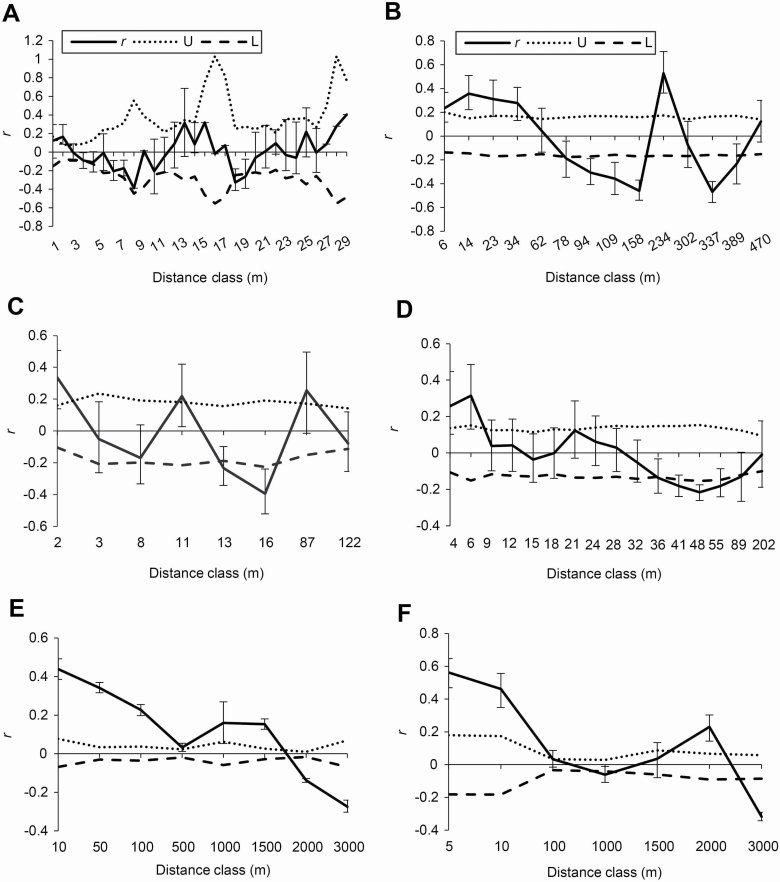
Correlogram showing the spatial autocorrelation coefficient (*r*) within subpopulations in metapopulations of *Zingiber corallinum* and *Z*. *nudicarpum*. U and L represent the 95 % two-tailed confidence interval, which was calculated based on 999 permutations; (A–D) subpopulations 1–4 in the GDYX metapopulation of *Z*. *corallinum*; (E and F) subpopulations 1–2 in the HNCJ metapopulation of *Z*. *nudicarpum*.

**Figure 7.**
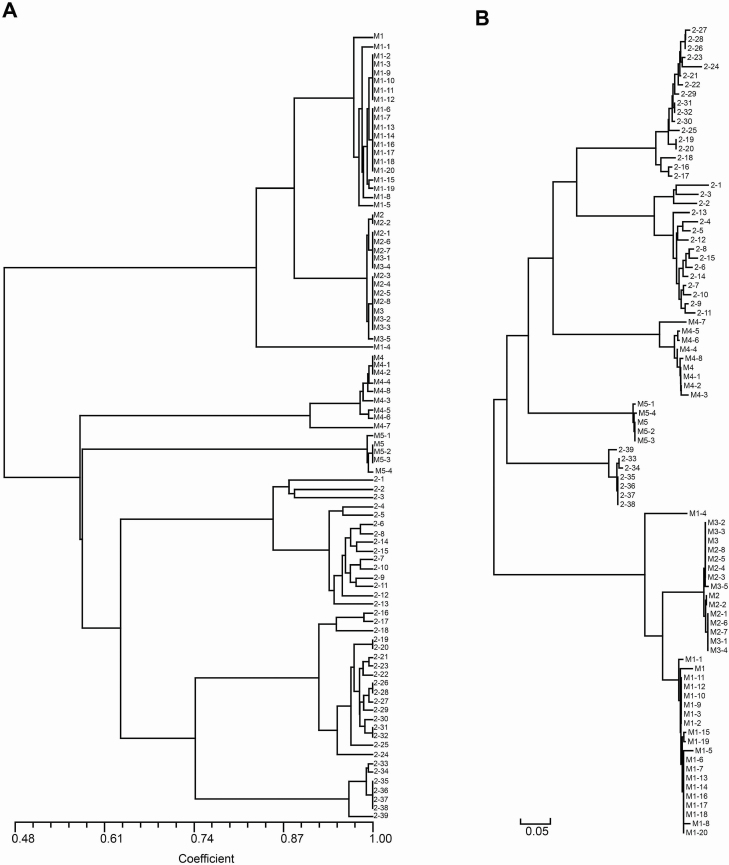
UPGMA dendrogram (A) based on Dice coefficient and unrooted NJ tree (B) based on Nei’s genetic distance for 45 seedlings and all adults within the GDZJ-2 subpopulation in the GDZJ metapopulation of *Zingiber corallinum* (M1–M5, potential maternal plants; M1-1–M5-4, seedlings; 2-1–2-39, other adult plants).

The spatial autocorrelation analysis indicated that significant positive spatial genetic structure was detected at 2–34 m (*r* = 0.282 ± 0.058, *P* < 0.05) and 100–1500 m (*r* = 0.268 ± 0.181, *P* < 0.05) in subpopulations of selfing *Z*. *corallinum* metapopulation GDYX and outcrossing *Z*. *nudicarpum* metapopulation HNCJ, respectively (Fig. 6). Furthermore, the cluster analysis for seedlings and adults from subpopulation 2 in metapopulation in GDZJ revealed that, except for one seedling, all seedlings clustered with the nearest adult together as a clade (Fig. 7). The UPGMA dendrogram (Fig. 4) and the NJ tree **[see**  **Supporting Information—Fig. S3****]** also showed that, except for four individuals in the GDZJ metapopulation, all the neighbouring individuals within subpopulations grouped together in selfing *Z*. *corallinum* metapopulations. However, within outcrossing *Z*. *nudicarpum* subpopulations, although the majority of neighbouring individuals also clustered together, there were many individuals that did not cluster with their neighbours (Fig. 5; **see**  **Supporting Information—Fig. S4**). In addition, mantel tests showed that there was no significant isolation-by-distance relationship across subpopulations within metapopulations of *Z*. *corallinum* (GDZJ: *r* = 0.068, *P* = 0.545; GDYX: *r* = 0.000, *P* = 0.570) and outcrossing *Z. nudicarpum* metapopulation HNBT (*r* = 0.917, *P* = 0.161) **[see**  **Supporting Information—Fig. S6****]**.

## Discussion

### The differences in effects of habitat fragmentation on genetic diversity within metapopulations between selfing and outcrossing *Zingiber* species

Compared to selfing species, genetic diversity within populations of outcrossing species tends to be higher and differentiation among populations tends to be lower (Clasen *et al.* 2011). However, our data revealed that although the level of subpopulation genetic diversity in selfing *Z*. *corallinum* was significantly lower than that in outcrossing *Z*. *nudicarpum* (*h* = 0.0662 vs. 0.1464, *P* = 0.028; *I* = 0.0995 vs. 0.2257, *P* = 0.023), the level of metapopulation genetic diversity of selfing *Z*. *corallinum* was comparable to that of outcrossing *Z*. *nudicarpum* (*h* = 0.2490 vs. 0.2246, *P* = 0.295; *I* = 0.3753 vs. 0.3480, *P* = 0.438). The mating system (i.e. selfing vs. outcrossing) can strongly influence the vulnerability to fragmentation effects on genetic diversity (Aguilar *et al.* 2008). Although populations of outcrossing species can maintain a high level of genetic diversity through frequent exchange of genes with other populations (Honnay and Jacquemyn 2007), sudden decreases in effective population sizes due to habitat fragmentation have strong negative effects on within-population genetic diversity of outcrossing species (Aguilar *et al.* 2008), such as tropical *Ficus* species with specialized pollinator systems (Nason and Hamrick, 1997), wind-pollinated outcrossing *Fagus sylvatica* (Jump and Peñuelas 2006) and insect-pollinated outcrossing *Lepidium subulatum* (Gómez-Fernández *et al.* 2016). Because of increasing habitat destruction and decreasing local population size, the exchange of alleles may be reduced, and genetic diversity may decrease without the possibility of replenishing the alleles (Honnay and Jacquemyn 2007). On the other hand, with severe inbreeding depression, inbred individuals harbouring deleterious alleles may die or not reproduce, effectively removing these alleles from the population. Thus, inbreeding will tend to purge populations of enough deleterious recessive mutations to reduce inbreeding depression (Crnokrak and Barrett 2002). Therefore, outcrossing species show stronger negative effects of fragmentation on genetic diversity than selfing species (Aguilar *et al.* 2008). In contrast, the level of population genetic diversity of mainly selfing species will be less affected by reduced gene flow because each individual contains most of the genetic diversity of the population (Honnay and Jacquemyn 2007).

Without migration among demes of subpopulations in metapopulations of selfing species, any mutation that arises in a particular subpopulation may be fixed in that subpopulation and cannot spread to other subpopulations. Therefore, compared to a large primary population, while an individual small population fragment may become homozygous for a particular allele, the overarching metapopulation could still maintain significant genetic diversity because the various population fragments it encompasses may fix different loci (Frankham *et al.* 2002). This is may be the case for selfing *Z*. *corallinum* in our study. The proportion of common loci within subpopulations of selfing *Z. corallinum* metapopulations was significantly higher than that in outcrossing *Z*. *nudicarpum* (67.6 % vs. 37.7 %, *P* = 0.041). However, both species contained similar levels of common loci in metapopulations (15.8 % vs. 16.2 %, *P* = 0.982). In addition, the specific band number within subpopulations and metapopulations of selfing *Z. corallinum* was higher than that in outcrossing *Z. nudicarpum*, but not significant (11.3 vs. 5, *P* = 0.114 and 39.5 vs. 12.5, *P* = 0.258, respectively). Together, these results imply that local adaptation and/or neutral mutation may have caused differentiation among subpopulations (patches) by fixation of different loci (Owuor *et al.* 1999) in selfing *Z*. *corallinum* metapopulations. The increased diversity between populations is paralleled by a similarly high level of diversity between allelic classes at polymorphic loci, which have very different allele frequencies among subpopulations, thus resulting in a high level of diversity in the respective metapopulations (Charlesworth *et al.* 1997). Due to the lack of gene flow and homogeneous habitat, it is not likely that local adaptation (nature selection) may have contributed significantly to the increased genetic differentiation between subpopulations within *Z*. *corallinum* metapopulations. The Mantel tests also show selfing *Z*. *corallinum* does not exhibit a pattern of isolation by distance among subpopulations within metapopulations, suggesting that the stochastic force of genetic drift is much stronger than gene flow in determining the structure of subpopulations (Pettengill *et al.* 2016) within selfing *Z*. *corallinum* metapopulations. Here, we suggest that genetic diversity of selfing *Z*. *corallinum* can be maintained at the metapopulation level due to differentiation among subpopulations and that the genetic variability among subpopulations is expected to increase continuously with time, due to new mutations adding in continuously. Compared with the landscape level, selfing *Z. corallinum* could maintain high genetic diversity through differentiation intensified primarily by the stochastic force of genetic drift among subpopulations at fine-scale level, but not local adaptation.

### Genetic structure patterns within metapopulations of selfing and outcrossing species

Outcrossing plants typically show higher genetic variation within populations or subpopulations, whereas in selfing plants most of the genetic variation is found among populations or subpopulations (Honnay and Jacquemyn 2007). Our AMOVA analysis also revealed that the major portion of genetic variation in selfing *Z*. *corallinum* metapopulations resides among subpopulations (66.3–90.5 %), while a lower degree of genetic variance (9.5–33.7 %) exists within different subpopulations. However, the majority of variation was also found among subpopulations (62.5 %), rather than within subpopulations (37.5 %) in outcrossing *Z*. *nudicarpum* metapopulation HNCJ, in which gene flow was seriously eroded by habitat fragmentation (*N*_m_ = 0.7180 < 1) and genetic differentiation among subpopulations was higher. The opposite was true in the outcrossing *Z*. *nudicarpum* metapopulation HNBT, in which gene flow was not significantly affected by habitat fragmentation (*N*_m_ = 1.9734 > 1). The genetic structure of outcrossing *Z*. *nudicarpum* metapopulations can be attributed to the short distances of pollen movement via parasitic bees and the constraints of seed dispersal by gravity. In the metapopulation HNBT, three subpopulations scattered widely in a more or less continuous area of suitable habitat, which separated by 200–550 (average ca. 320) m apart. This isolation degree could not prevent the pollen movement of outcrossing *Z. nudicarpum* between populations, as evidenced by the significant positive autocorrelation of spatial genetic structure with 100–1500 m. However, the two subpopulations in the metapopulation HNCJ are isolated by 450–1000 (average ca. 725) m of mountain forest. This greater isolation could significantly prevent the pollen migration of outcrossing *Z. nudicarpum* between populations. In comparison, the genetic structure of selfing *Z*. *corallinum* metapopulations is influenced almost entirely by restricted seed dispersal due to gravity alone. Our results suggest that the majority of genetic variation resides among subpopulations in selfing *Z*. *corallinum* metapopulations, while the major portion of genetic variation exists within or among subpopulations in outcrossing *Z*. *nudicarpum* metapopulations, most probably depending on whether the degree of subpopulation isolation surpasses the dispersal ability of pollen and seed.

Our cluster analysis showed that neighbouring individuals within subpopulations always grouped together in selfing *Z*. *corallinum* metapopulations and all seedlings also clustered with their nearest adults. Moreover, the significant positive autocorrelation of spatial genetic structure occurs within only 2–34 m in subpopulations of selfing *Z*. *corallinum*. The above autocorrelations over short distances reflect the occurrence of patches of genetically similar individuals (Torres *et al.* 2003) in selfing *Z*. *corallinum* metapopulations. Previous studies have also shown that a high level of spatial genetic structure is typical of a population of predominantly selfing and gravity-dispersed plants (Volis *et al.* 2010, 2016; Barluenga *et al.* 2011). For selfing *Z*. *corallinum* metapopulations, this is the logical consequence of two phenomena, the high levels of self-fertilization leading to inflated inbreeding in the offspring. Restricted gravity-driven seed dispersal around the parents could cause aggregated distribution of offspring in maternal half-sib families or full-sib families (Bittencourt and Sebbenn 2007). However, many individuals did not aggregate with their neighbours within subpopulations in outcrossing *Z*. *nudicarpum* metapopulations, and significant positive autocorrelation of spatial genetic structure occurred over distances of 100–1500 m. This is consistent with the hypothesis that outcrossing species always tend to generate a lower spatial genetic structure than selfing species (Vekemans and Hardy 2004), presumably due to higher gene flow via pollen (Duminil *et al.* 2009). In outcrossing plant species, pollen dispersal contributes to overall gene dispersal, whereas in highly selfing species, seed dispersal alone governs overall gene dispersal (Vekemans and Hardy 2004). Genetic analyses using the STRUCTURE software package also showed that all individuals within subpopulations in outcrossing *Z*. *nudicarpum* metapopulation HNCJ (*N*_m_ = 0.7180 < 1) were assigned to the same genetic cluster, but this was not the case in metapopulation HNBT (*N*_m_ = 1.9734 > 1). Moreover, UPGMA and NJ analysis revealed that all individuals within subpopulations in metapopulation HNCJ were clustered together as a single clade, but, again, metapopulation HNBT did not conform to this pattern. The results of the PCoA revealed a similar clustering pattern.

In summary, our results indicate that restricted gene flow as a result of gravity-driven seed dispersal contributes to the genetic differentiation between subpopulations or fragments within metapopulations of selfing *Z*. *corallinum*. Although limited seed dispersal may have a stronger effect on the genetic structure of a population, pollen movement could promote gene exchange between or within subpopulations or fragments within outcrossing *Z. nudicarpum* metapopulations. Thus, contrary to our expectations, a weaker genetic structure appears in species like *Z. nudicarpum* with extensive pollen movement but restricted seed dispersal when such species occur in fragmented habitats.

## Supporting Information

The following additional information is available in the online version of this article—

Figure S1. Plant, inflorescence and flower of *Zingiber corallinum* (A) and *Z*. *nudicarpum* (B).

Figure S2. Mean log-likelihood probability of data Ln*P* (*K*) and Δ*K* estimates (A—HNCJ; B—HNBT).

Figure S3. Unrooted neighbour-joining trees based on Nei’s genetic distance for individuals in metapopulations of *Zingiber corallinum* (A—GDZJ; B—GDYX).

Figure S4. Unrooted neighbour-joining trees based on Nei’s genetic distance for individuals in metapopulations of *Zingiber nudicarpum* (A—HNCJ; B—HNBT).

Figure S5. Scatterplot of the principal coordinate analysis (PCoA) based on inter-simple sequence repeat (ISSR) polymorphisms for individuals in metapopulations of *Zingiber corallinum* (A—GDZJ; B—GDYX) and *Z. nudicarpum* (C—HNCJ, D—HNBT).

Figure S6. Correlation between geographical distance and Nei’s genetic distance among subpopulations within metapopulations of *Zingiber corallinum* (A—GDZJ; B—GDYX) and Z. nudicarpum (C—HNBT).

Table S1. The 64 inter-simple sequence repeat (ISSR) primers used previously in references for Zingiberaceae.

Table S2. Attributes of inter-simple sequence repeat (ISSR) primers of *Zingiber corallinum* (ZC) and *Z*. *nudicarpum* (ZN) used in the present study.

plaa065_suppl_Supplementary_Figures_and_Tables

plaa065_suppl_Supplementary_Material

## Data Availability

Inter-simple sequence repeat (ISSR) data for *Z*. *corallinum* and *Z*. *nudicarpum* are available as Supporting Information.

## Notes

**Evolution & Diversity. Chief Editor:** Jeremy Beaulieu
