# Supplementary material for: Genetic variation and genetic structure within metapopulations of two closely related selfing and outcrossing Zingiber species (Zingiberaceae)
Source: AoB Plants. 2020 Dec 2;13(1):plaa065. doi: 10.1093/aobpla/plaa065 (PMC7788390; doi:10.1093/aobpla/plaa065)
Supplement: plaa065_suppl_Supplementary_Figures_and_Tables [file plaa065_suppl_supplementary_figures_and_tables.pdf]

Supporting Information Figure S1

Plant, inflorescence and flower of *Zingiber corallinum* (A) and *Z. nudicarpum* (B).

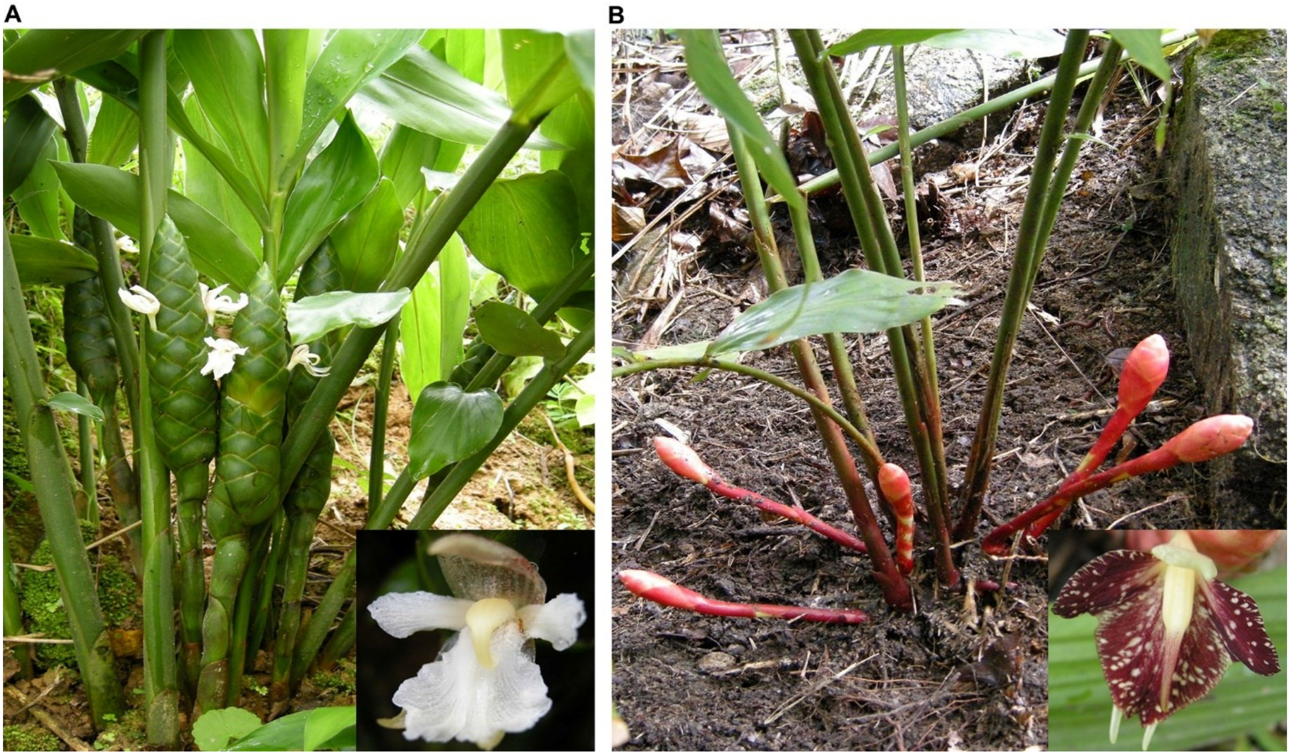

Supporting Information Figure S2

Mean log-likelihood probability of data LnP (K) and ΔK estimates (A--HNCJ, B—HNBT).

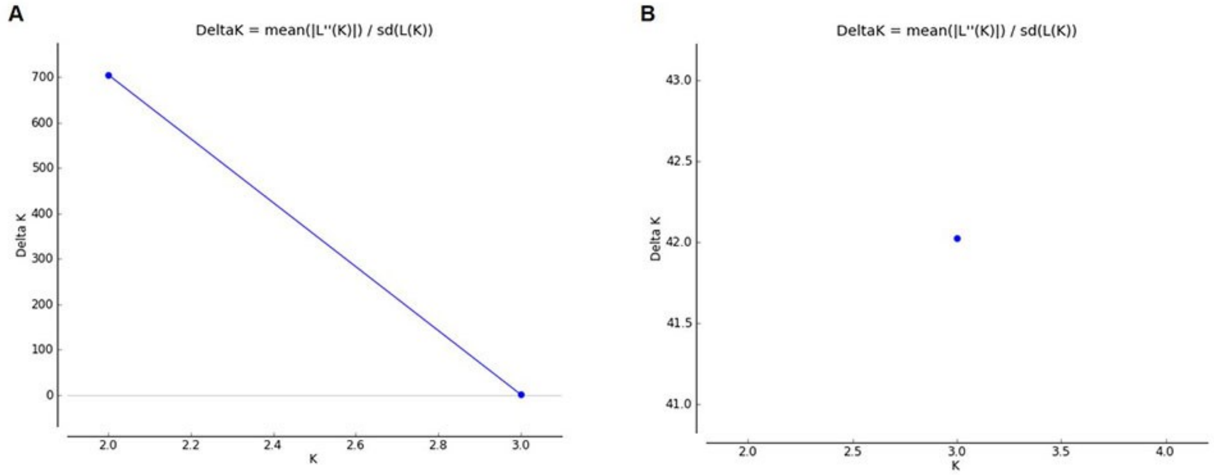

Supporting Information Figure S3

Unrooted Neighbor-Joining trees based on Nei's genetic distance for individuals in metapopulations of *Zingiber corallinum* (A--GDZJ, B--GDYX).

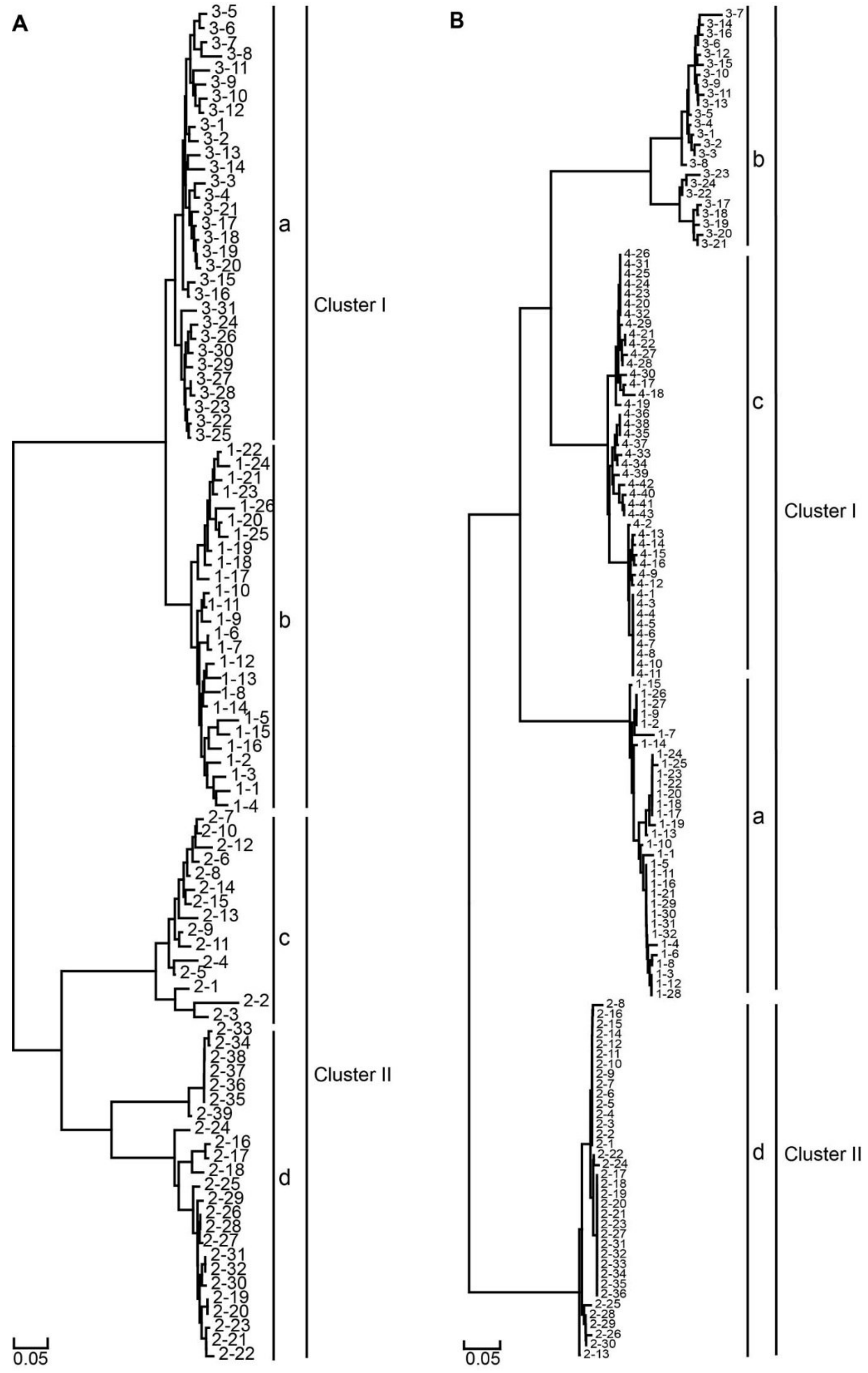

Supporting Information Figure S4

Unrooted Neighbor-Joining trees based on Nei's genetic distance for individuals in metapopulations of *Zingiber nudicarpum* (A--HNCJ, B--HNBT).

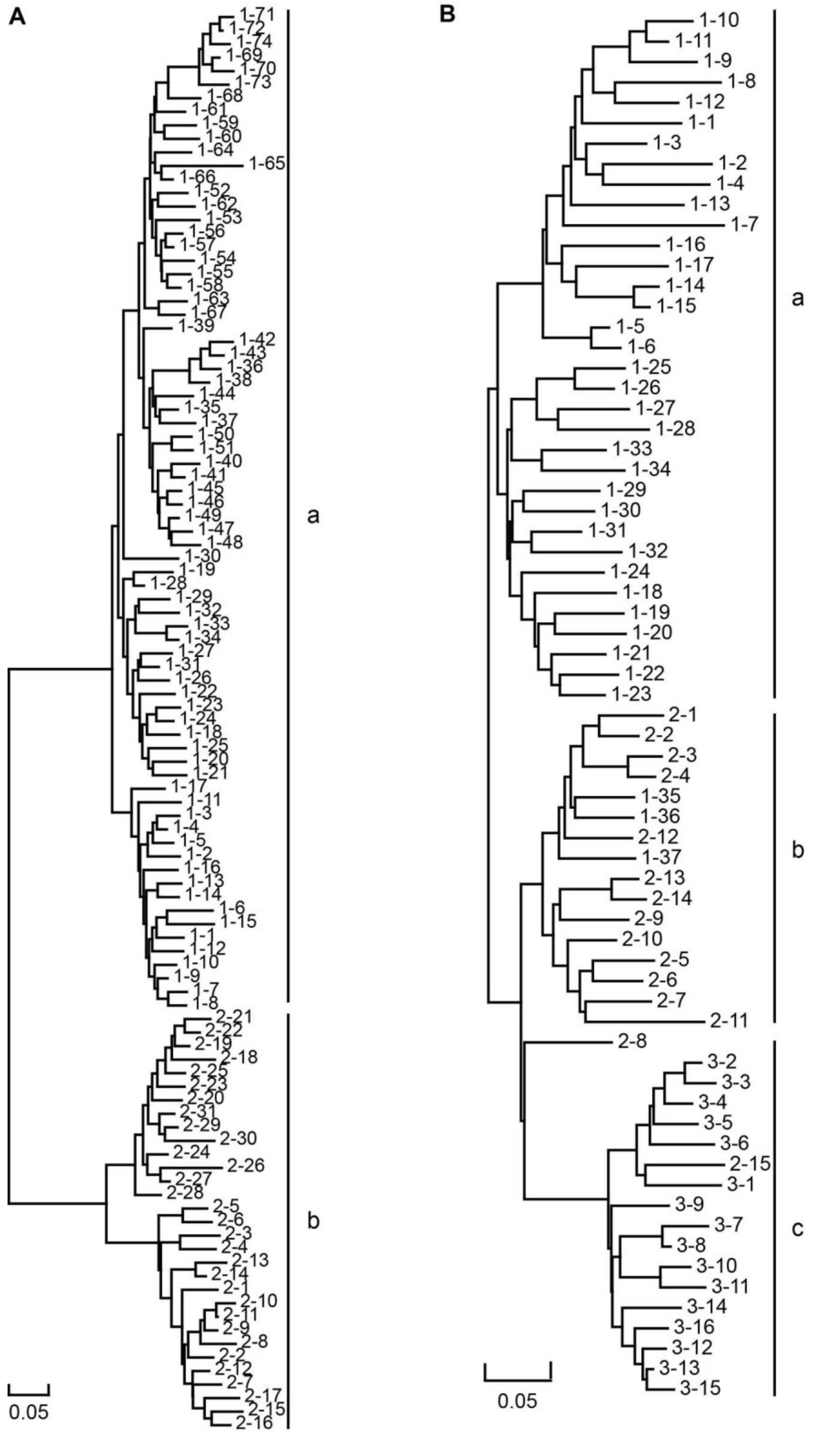

Supporting Information Figure S5

Scatterplot of the principal coordinate analysis (PCoA) based on ISSR polymorphisms for individuals in metapopulations of *Zingiber corallinum* (A--GDZJ, B--GDYX) and *Z. nudicarpum* (C--HNCJ, D--HNBT). Each number (1-4) represents one subpopulation while different colors represent individuals from different subpopulations.

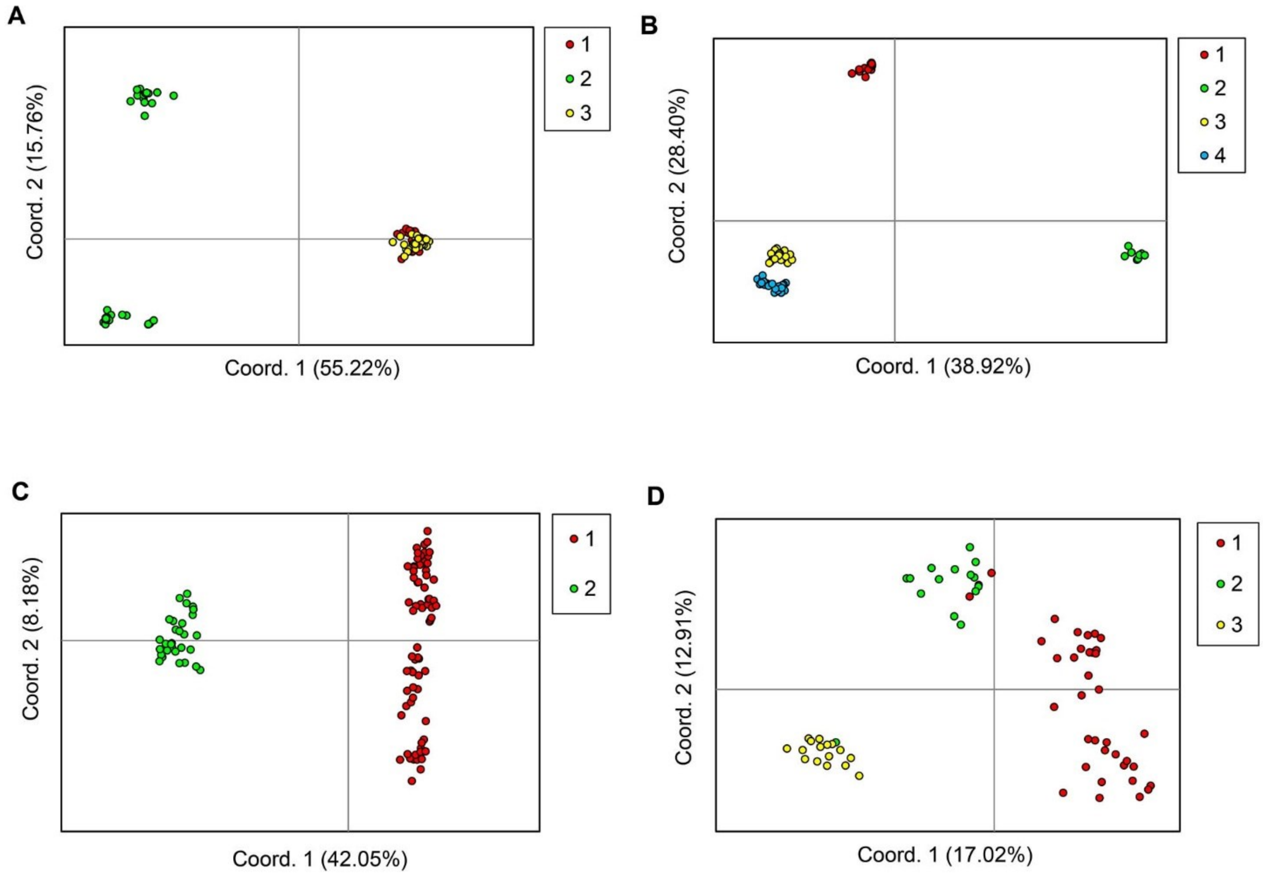

Supporting Information Figure S6

Correlation between geographical distance and Nei's genetic distance among subpopulations within metapopulations of *Zingiber corallinum* (A--GDZJ; B--GDYX) and *Z. nudicarpum* (C--HNBT).

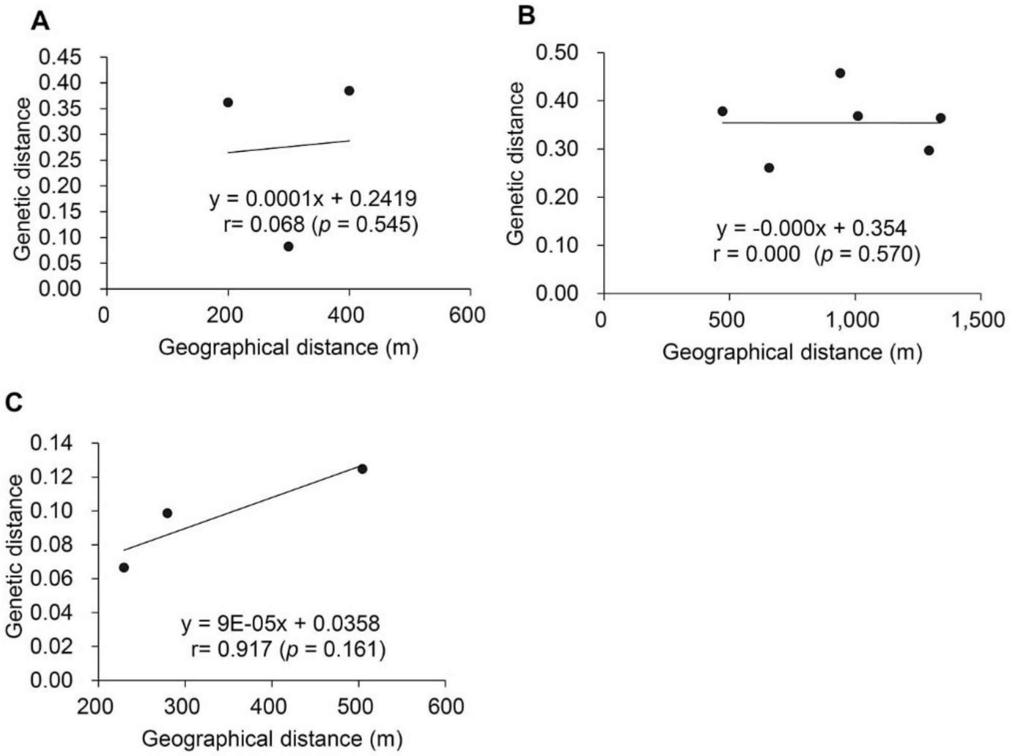

Supporting Information Table S1

The 64 ISSR primers used previously in references for Zingiberaceae.

Species	Sequence 5′ to 3′	Primer	Reference
Globba lancangensis<br>*C. amada*<br>*C. angustifolia*<br>*C. caesia*<br>*C. zedoaria*<br>*C. aromatic*<br>*C. longa*<br>*C. domestica*<br>*C. alismatifolia*<br>*C. wenyujin*<br>*Alpinia oxyphylla*<br>*Curcuma wenyujin*<br>*Zingiber moran*	(AG)8 T	807	Zhou HP, Chen J, Chen F. 2007. Ant-mediated seed dispersal contributes to the local spatial pattern and genetic structure of *Globba lancangensis* (Zingiberaceae). *Journal of Heredity* **98**: 317-324.<br>Das A, Kesari V, Madurai Satyanarayana V, Parida A, Rangan L. 2011. Genetic relationship of *Curcuma* species from Northeast India using PCR-based markers. *Molecular Biotechnology* **49**: 65-76.<br>Taheri S, Abdullah TL, Abdullah NAP, Ahmad Z. 2012. Genetic relationships among five varieties of *Curcuma alismatifolia* (Zingiberaceae) based on ISSR markers. *Genetics and molecular research* **11**: 3069-3076.<br>Wang HY, Liu XJ, Wen MF, Pan K, Zou ML, Lu C, Liu SS, Wang WQ. 2012. Analysis of the genetic diversity of natural populations of *Alpinia oxyphylla* Miquel using Inter-Simple Sequence Repeat Markers. *Crop Science* **52**: 1767-1775.<br>Zheng WH, Zhuo Y, Liang L, Ding WY, Liang LY, Wang XF. 2015. Conservation and population genetic diversity of *Curcuma wenyujin* (Zingiberaceae), a multifunctional medicinal herb. *Genetics and molecular research* **14**: 10422-10432.<br>Das A, Kesari V, Satyanarayana VM, Parida A, Mitra S, Rangan L. 2015. Genetic diversity in ecotypes of the scarce wild medicinal crop *Zingiber moran* revealed by ISSR and AFLP marker analysis and chromosome number assessment. *Plant Biosystems* **149**: 111-120.
	(AG)8 C	808	
	(GA)8 T	810	
	(TC)8 G	824	
	(AC)8 G	827	
	(AG)8 Y†C	835	
	(GA)8 Y†T	840	
	(AC)8 Y†T	855	
	(AC)8 Y†G	857	
	VDV† (TC)7	886	
	DVD† (TC)7	887	
	BDB† (CA)7	888	
	BDB† (AC)7	889	
	VHV† (GT)7	890	

Table S1. (continued).

Species	Sequence 5′ to 3′	Primer	Reference
Curcuma phaeocaulis	(AG)8G	809	Wang XH, Tang XC, Yang EX, Liu DJ, Li M, Li XK. 2008. ISSR-PCR analysis in different species and populations of Rhizoma *Curcuma*. *China Journal of Chinese Materia Medica* **18**: 2037-2040.
C. kwangsiensis	(AC)8C	862	
C. wenyujin	(ACC)6	861	
C. amada	(GAA)6	868	
C. angustifolia	(GACA)4	873	
C. caesia			
C. zedoaria			Das A, Kesari V, Madurai Satyanarayana V, Parida A, Rangan L. 2011. Genetic relationship of *Curcuma* species from Northeast India using PCR-based markers. *Molecular Biotechnology* **49**: 65-76.
C. aromatic			
C. longa			
C. domestica			
Alpinia oxyphylla			
Zingiber moran			
			Wang HY, Liu XJ, Wen MF, Pan K, Zou, ML, Lu C, Liu SS, Wang WQ. 2012. Analysis of the genetic diversity of natural populations of *Alpinia oxyphylla* Miquel using Inter-Simple Sequence Repeat Markers. *Crop Science* **52**: 1767-1775.
C. amada	(AG)8C	811	Taheri S, Abdullah TL, Abdullah NAP, Ahmad Z. 2012. Genetic relationships among five varieties of *Curcuma alismatifolia* (Zingiberaceae) based on ISSR markers. *Genetics and molecular research* **11**: 3069-3076.
C. angustifolia	(CA)8T	816	
C. caesia	(CA)8A	817	
C. zedoaria	(CA)8G	818	
C. aromatic	(GT)8A	819	
C. longa	(AC)8T	825	
C. domestica	(AC)8C	826	Wang HY, Liu XJ, Wen MF, Pan K, Zou ML, Lu C, Liu SS, Wang WQ. 2012 Analysis of the genetic diversity of natural populations of *Alpinia oxyphylla* Miquel using Inter-Simple Sequence Repeat Markers. *Crop Science* **52**: 1767-1775.
C. alismatifolia	(CT)8AC	844	
C. wenyujin	(AG)8Y†A	837	
Alpinia oxyphylla	(TA)8R†C	838	
Zingiber moran	(GATA)4	872	
			Das A, Kesari V, Satyanarayana VM, Parida A, Mitra S, Rangan L. 2015. Genetic diversity in ecotypes of the scarce wild medicinal crop *Zingiber moran* revealed by ISSR and AFLP marker analysis and chromosome number assessment. *Plant Biosystems* **149**: 111-120.

Table S1. (continued).

Species	Sequence 5′ to 3′	Primer	Reference
Curcuma alismatifolia *Alpinia oxyphylla* *Curcuma wenyujin*	(GA)8A	812	Taheri S, Abdullah TL, Abdullah NAP, Ahmad Z. 2012. Genetic relationships among five varieties of *Curcuma alismatifolia* (Zingiberaceae) based on ISSR markers. *Genetics and molecular research*, **11**: 3069-3076. Wang HY, Liu XJ, Wen MF, Pan K, Zou ML, Lu C, Liu SS, Wang WQ. 2012. Analysis of the genetic diversity of natural populations of *Alpinia oxyphylla* Miquel using Inter-Simple Sequence Repeat Markers. *Crop Science* **52**: 1767-1775. Zheng WH, Zhuo Y, Liang L, Ding WY, Liang LY, Wang XF. 2015. Conservation and population genetic diversity of *Curcuma wenyujin* (Zingiberaceae), a multifunctional medicinal herb. *Genetics and molecular research* **14**: 10422-10432.
	(TG)8A	828	
	(AG)8Y†T	834	
	(GA)8Y†	841	
	(GA)8Y†G	842	
	(CA)8R†C	847	
	(CA)8R†G	848	
	(GT)8Y†C	850	
	(GGAGA)3	880	
Alpinia oxyphylla *Curcuma wenyujin*	(CT)8T	813	Wang HY, Liu XJ, Wen MF, Pan K, Zou ML, Lu C, Liu SS, Wang WQ. 2012. Analysis of the genetic diversity of natural populations of *Alpinia oxyphylla* Miquel using Inter-Simple Sequence Repeat Markers. *Crop Science* **52**: 1767-1775. Zheng WH, Zhuo Y, Liang L, Ding WY, Liang LY, Wang XF. 2015. Conservation and population genetic diversity of *Curcuma wenyujin* (Zingiberaceae), a multifunctional medicinal herb. *Genetics and molecular research* **14**: 10422-10432.
	(CT)8A	814	
	(TC)8A	822	
	(CT)8R†A	843	
	(ATG)6	864	
	GGG(TGGGG)2TG	881	
	HVH† (TG)7	891	
	CAT(GGT)2GGTCATTGTTCCA	899	

Table S1. (continued).

Species	Sequence 5′ to 3′	Primer	Reference
Curvuma wenyujin	(TG)8C	829	Lei YX. 2013. *Genetic diversity among Curcuma species based on ISSR and RAMP Markers*. Master's Thesis, Sichuan Agricultural University, Yaan.
C. kwangsiensis	(CA)8R†T	846	
C. aromatica	(TC)8R†T	853	
C. phaeocaulis	(AC)8Y†A	856	
C. yunnanensis	(GGAT)4	878	
C. longa	HBH† (AG)7	884	
C. sichuamnsis	BHB† (GA)7	885	
C. chuanhuangjiang			
A. villosum	(AG)8Y†A	836	Huang QL, Yang JF, Yan P, Zhan RT, Xu H, Chen WW. 2010. Establishment and Optimization of ISSR-PCR Reaction System for *Amomum villosum* Lour. *Lishizhen Medicine and Materia Medica Research* **10**: 2478-2480.
Curcuma wenyujin	(TG)8G	830	Zheng WH, Zhuo Y, Liang L, Ding WY, Liang LY, Wang XF. 2015. Conservation and population genetic diversity of *Curcuma wenyujin* (Zingiberaceae), a multifunctional medicinal herb. *Genetics and molecular research* **14**: 10422-10432.
	(CT)8R†G	845	
	(GT)8Y†A	849	
	(GT)8Y†G	851	
	(CT)8G	815	
	(TC)8T	823	
	(TG)8R†C	859	
	VBV† (AT)7	882	Tao ZM, Leng CH, Wu ZG, Li L. 2009. Analysis of genetic diversity in *Curcuma wenyujin* by ISSR. *Acta Agriculturae Zhejiangensis*, **21**: 207-210.
	ACTTCCACAGGTTAACACA	900	

†B = (C, G, T), D = (A, G, T), R = (A, T), V = (A, C, G), Y = (C, G), H = (A, C, T).

Supporting Information Table S2

Attributes of ISSR primers of *Zingiber corallinum* (ZC) and *Z. nudicarpum* (ZN) used in the present study.

Primer	Sequence 5′ to 3′	*T*m (°C)	SR (bp)	NT	NP	Species
807	(AG)8T	47	500-1500	15	15	ZN
808	(AG)8C	58	150-2000	24	23	ZN
810	(GA)8T	49 /53	200-1600/210-1600	22/23	20/20	ZC/ZN
811	(GA)8T	50/52	250-1800/250-1900	22/22	22/22	ZC/ZN
834	(AG)8Y†T	53/50	240-2150/250-1900	24/20	23/20	ZC/ZN
835	(AG)8Y†C	58	300-2000	24	24	ZN
841	(GA)8Y†C	55/52	230-1900/250-1900	23/21	22/21	ZC/ZN
847	(CA)8R†C	52/55	450-1600/400-1800	17/19	17/19	ZC/ZN
857	(AC)8Y†G	50/58	250-1800/240-2100	19/33	19/33	ZC/ZN
884	HBH†(AG)7	48/56	200-1900/220-1950	29/24	29/24	ZC/ZN
887	DVD†(TC)7	50/56	320-2100/390-2100	21/22	20/20	ZC/ZN
888	BDB†(CA)7	55/56	200-1700/240-1900	24/29	24/29	ZC/ZN
889	DBD†(AC)7	49/55	250-1900/210-1900	20/24	18/24	ZC/ZN
Total	–	–	200-2150/210-1900	221/300	215/294	ZC/ZN

†B = (C, G, T), D = (A, G, T), R = (A, T), V = (A, C, G), Y = (C, G), H = (A, C, T). *T*m: annealing temperature; SR: size range of amplified fragments; NT: total number of bands; NP: number of polymorphic bands.
